# Supplementary material for: Smart touchless human–machine interaction based on crystalline porous cages
Source: Nat Commun. 2024 Feb 21;15:1575. doi: 10.1038/s41467-024-46071-8 (PMC10881501; doi:10.1038/s41467-024-46071-8)
Supplement: Supplementary file 1 — Supplementary Information [file 41467_2024_46071_MOESM1_ESM.pdf]

Supplementary Information

Smart Touchless Human-Machine Interaction Based on Crystalline Porous Cages

*Jinrong Wang,[1] Weibin Lin,[1] Zhuo Chen,[2] Valeriia O. Nikolaeva,[1] Lukman O. Alimi,[1] Niveen M. Khashab[1,2]**

[1]Smart Hybrid Materials Laboratory (SHMs), Physical Science and Engineering Division, King Abdullah University of Science and Technology (KAUST), Thuwal 23955-6900, Saudi Arabia.

[2]Advanced Membranes and Porous Materials Center (AMPM), King Abdullah University of Science and Technology (KAUST), Thuwal 23955-6900, Saudi Arabia.

* Niveen M. Khashab. Email: niveen.khashab@kaust.edu.sa

Supplementary Figures

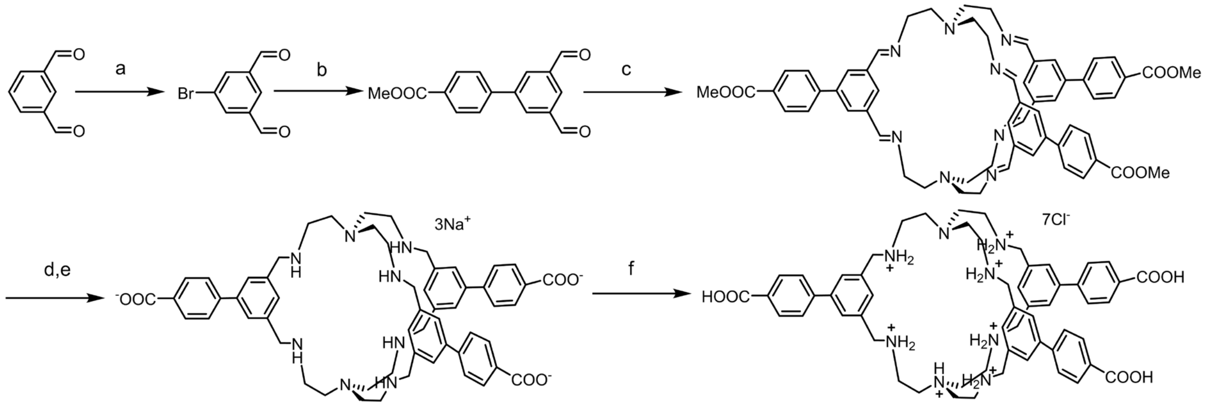

Supplementary Fig. 1 Synthetic route to prepare protonated Cage-1 Reagents and conditions: a). N-Bromosuccinimide, conc. H_2SO_4, reflux, 99%; b). 4-Methoxycarbonylphenylboronic acid, $Pd(PPh_3)_4$, K_2CO_3, THF/H_2O (*v*/*v*, 2:1), 80°C, 12h, 72%; c). tris(2-aminoethyl)amine, DCM/MeOH (*v*/*v*, 2:1), r.t.,6h,98%; d). $NaBH_4$, DCM/MeOH(*v*/*v*, 2:1), r.t., 6h; e) NaOH aqueous solution, MeOH, reflux, 12h; f). excess HCl solution, r.t. 96%.

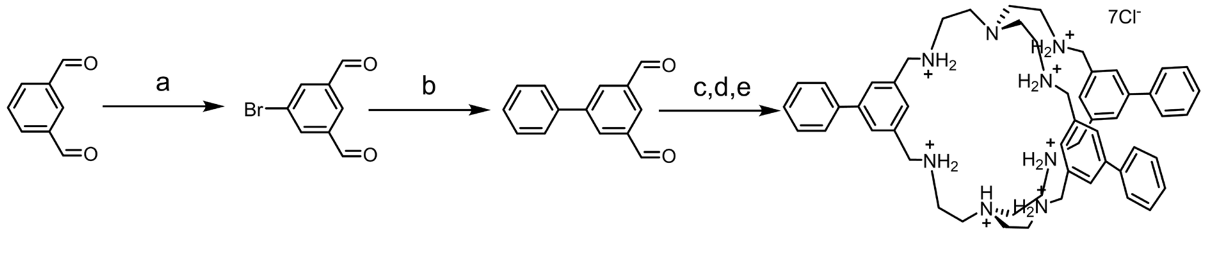

Supplementary Fig. 2 Synthetic route to prepare protonated Cage-2. Reagents and conditions: a) N-Bromosuccinimide, conc. H_2SO_4, reflux, 99%; b) 4-phenylboronic acid, $Pd(PPh_3)_4$, K_2CO_3, THF/H_2O (*v*/*v*, 2:1), 80°C, 12h, 75%; c) tris(2-aminoethyl)amine, DCM/MeOH (*v*/*v*, 2:1), r.t.,6h; d). $NaBH_4$, DCM/MeOH, r.t., 6h; e) EtOH, excess HCl solution, r.t. 91% for step c-e.

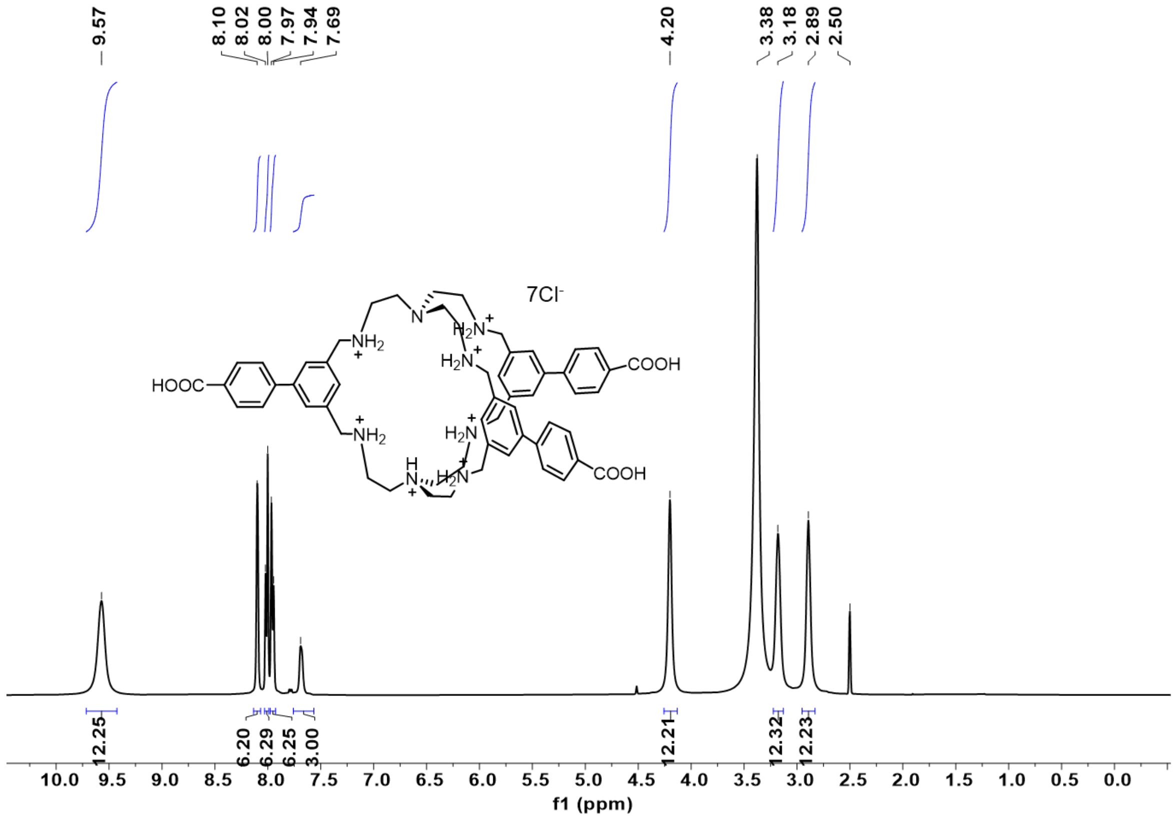

Supplementary Fig. 3 ^{1}H NMR spectrum (400 MHz, *d*-DMSO, 25 °C) of Cage-1.

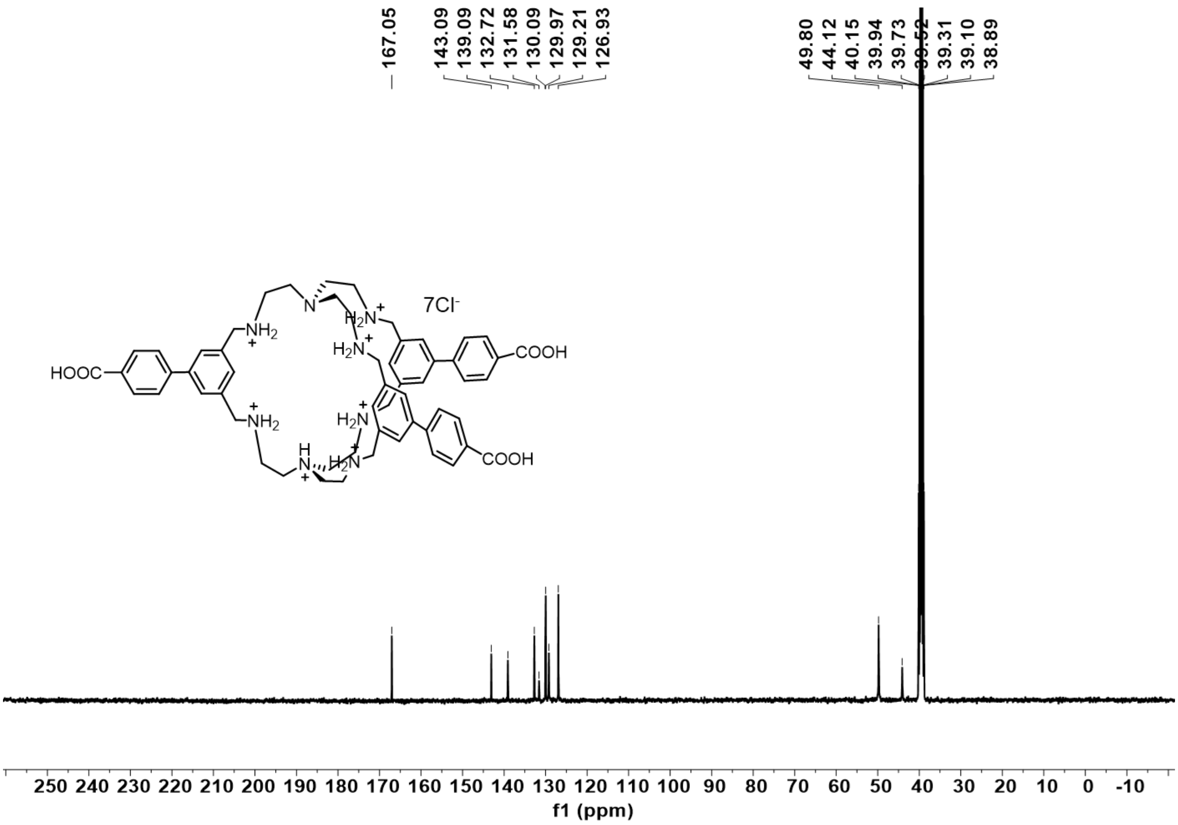

Supplementary Fig. 4 ^{13}C NMR spectrum (100 MHz, *d*-DMSO, 25 °C) of Cage-1.

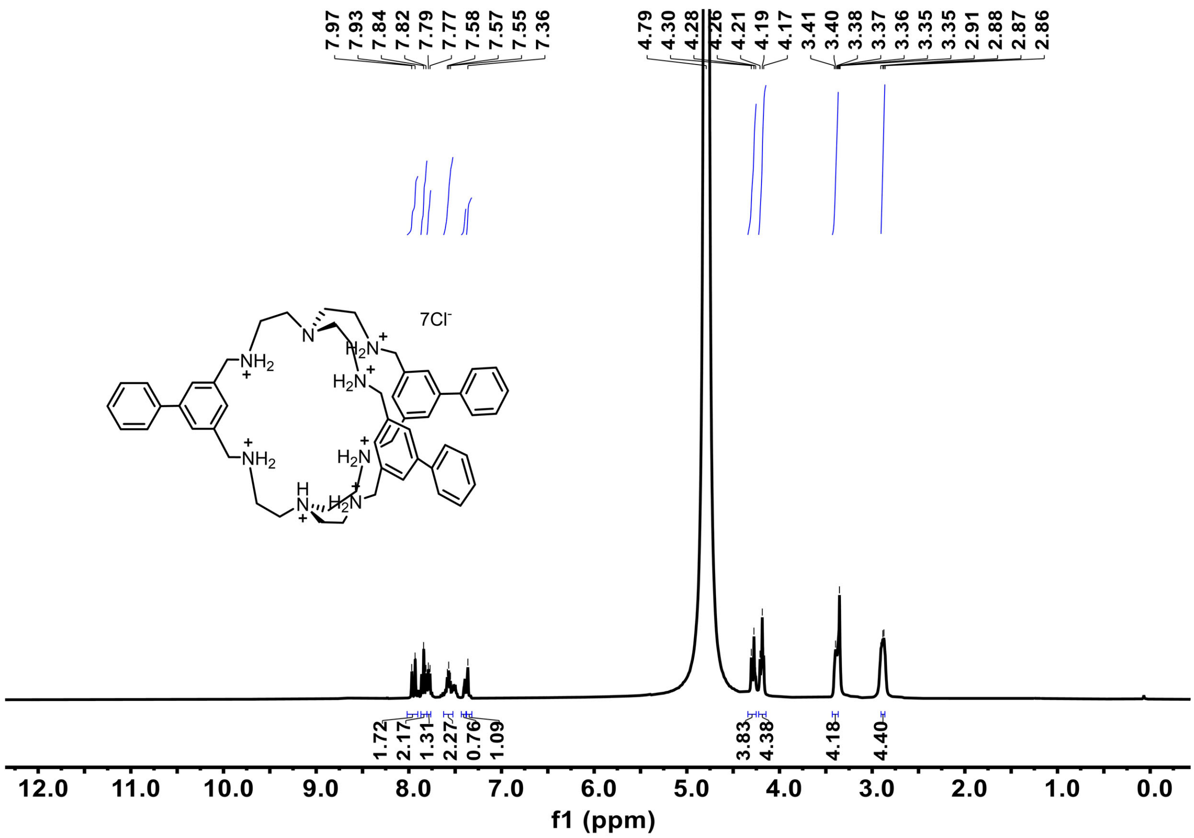

Supplementary Fig. 5 ^{1}H NMR spectrum (400 MHz, D_2O, 25 °C) of Cage-2.

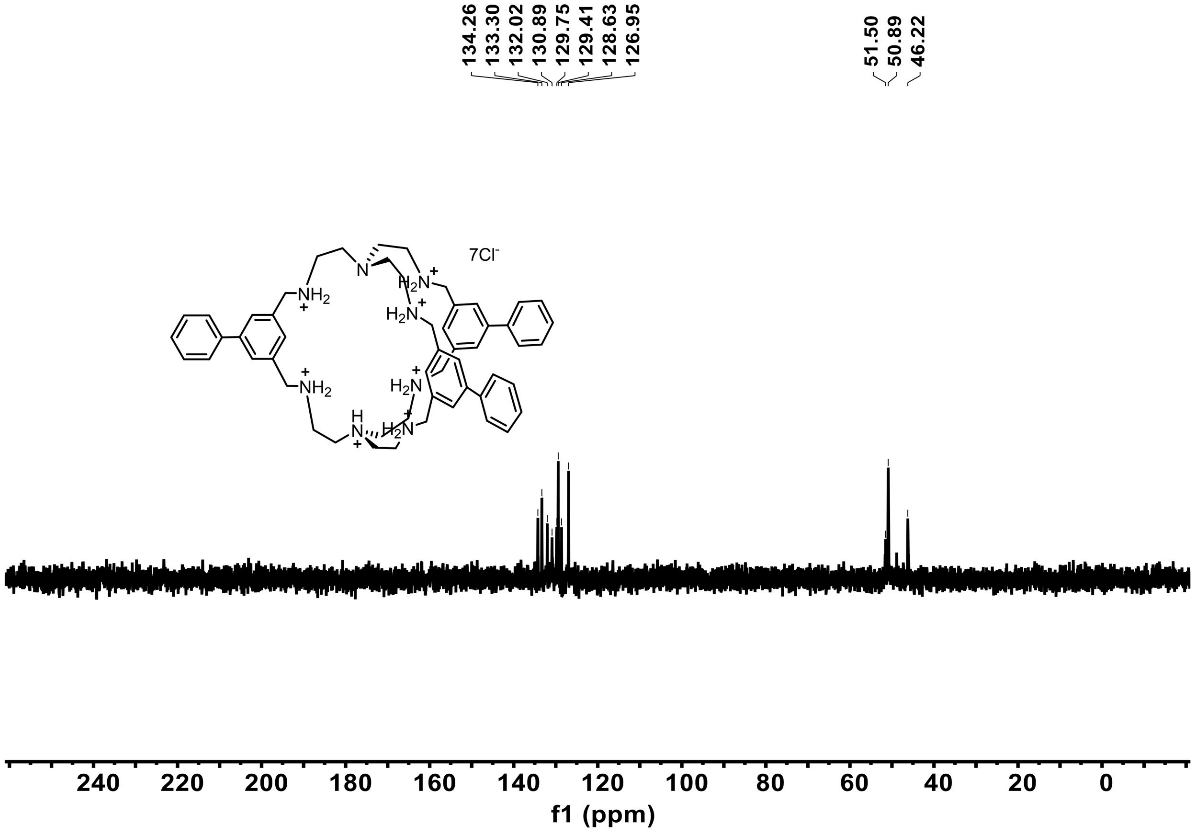

Supplementary Fig. 6 ^{13}C NMR spectrum (100 MHz, D_2O, 25 °C) of Cage-2.

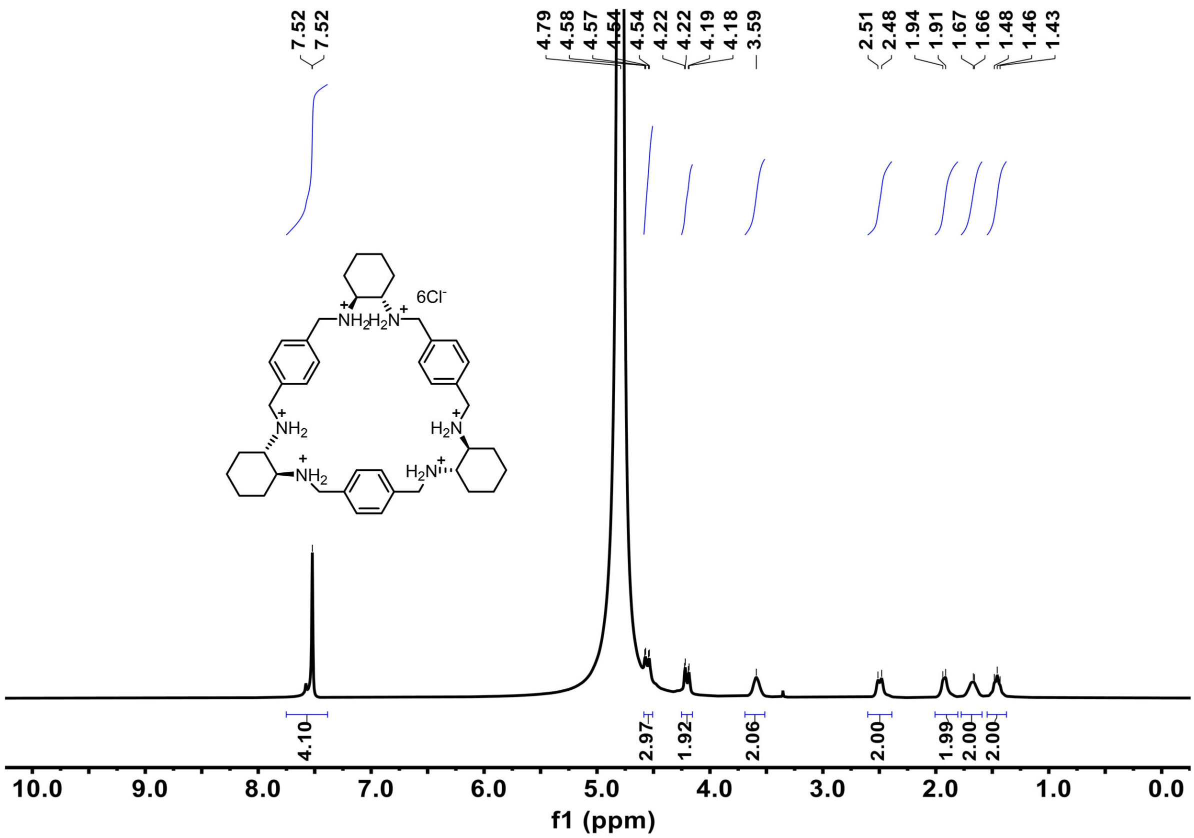

Supplementary Fig. 7 ^{1}H NMR spectrum (400 MHz, D_2O, 25 °C) of TA.

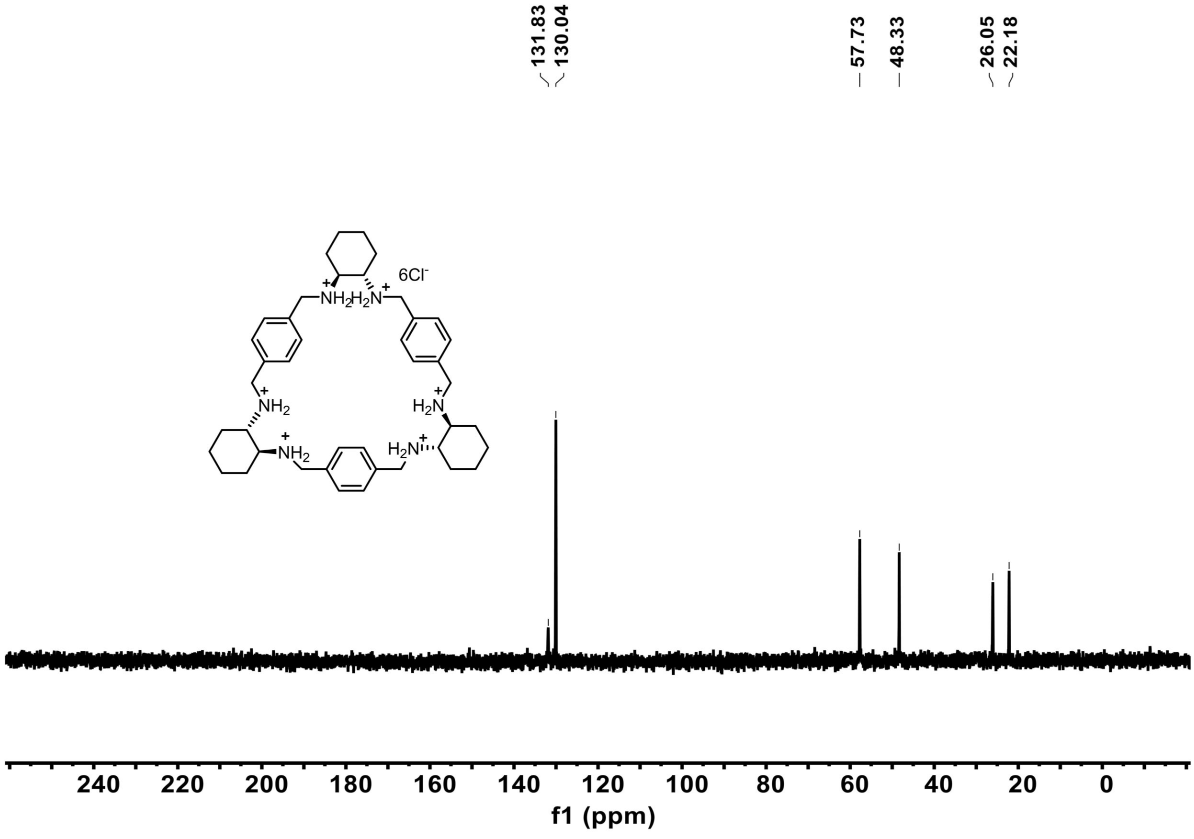

Supplementary Fig. 8 ^{13}C NMR spectrum (100 MHz, D_2O, 25 °C) of TA.

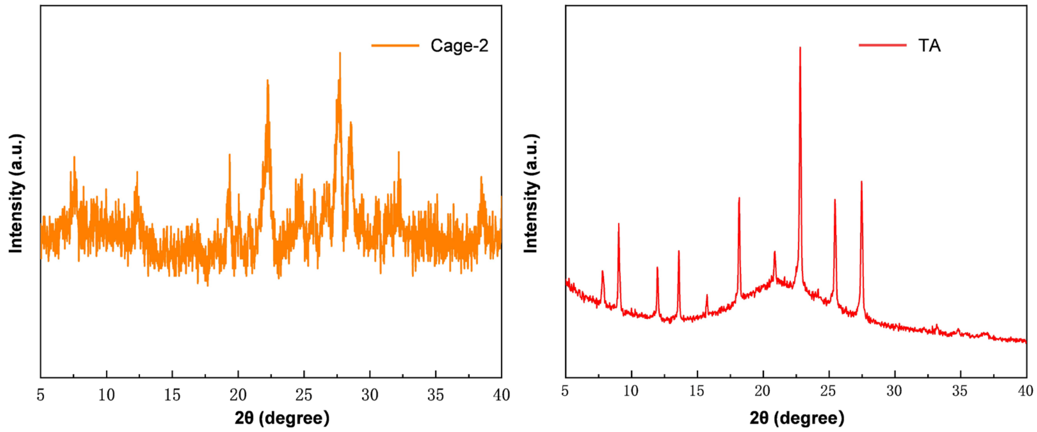

Supplementary Fig. 9 PXRD of the casted Cage-2 (left) and TA (right) sensing film.

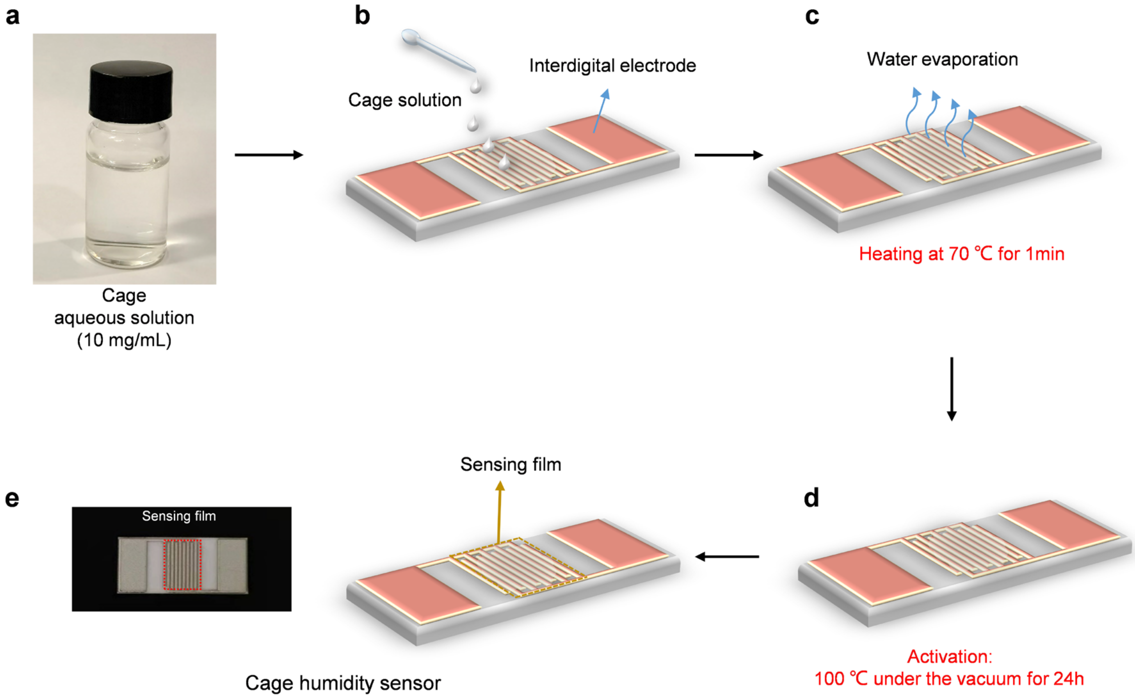

Supplementary Fig. 10 The fabrication process of the cage humidity sensor. (a) A 10mg/mL cage aqueous solution is prepared. **(b)** The solution is applied to the interdigital electrode surface through drop casting. **(c)** The coated surface is then heated at 70 °C for 1 minute. **(d)** Activation of the cage sensor takes place at 100 °C under vacuum conditions for 24 hours. **(e)** The final outcome is the cage sensor featuring a durable sensing film.

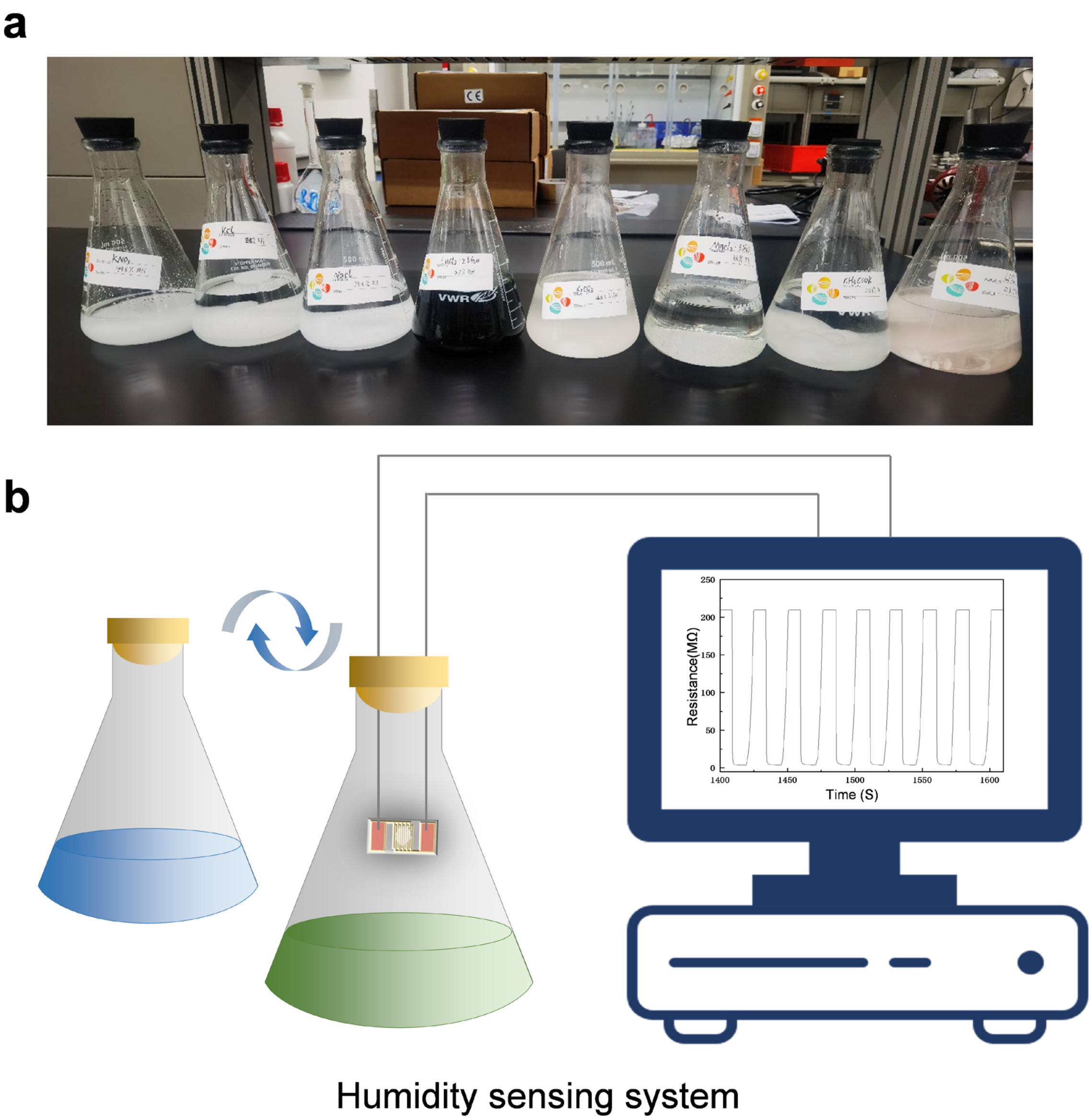

Supplementary Fig. 11 Humidity sensing test system. (a) Hermetic chambers with the desired RH are produced by various saturated salt solutions. **(b)** The humidity sensing system and sensing test method.

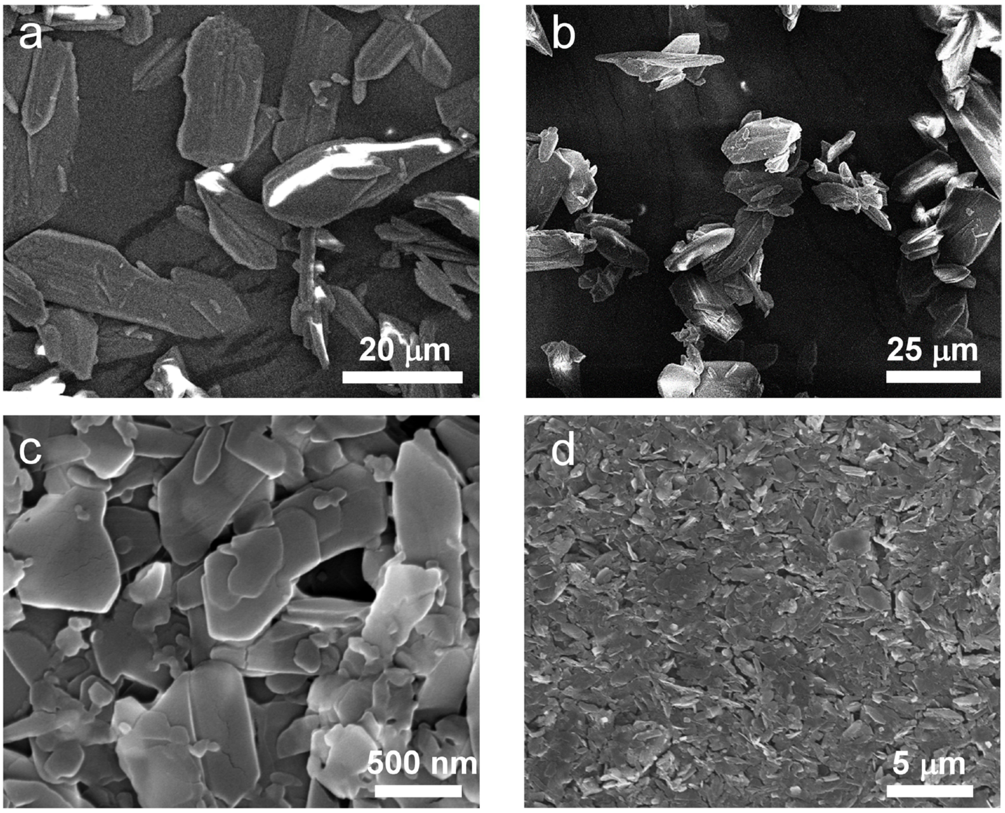

Supplementary Fig. 12 Structure comparison of cage powder and crystal sensing film. (a, b) SEM image of the as-prepared Cage-1 powder shows the large bulk structure sensing. **(c, d)** SEM image of the casted Cage-1 sensing film deposited on the interdigital electrode surface shows the submicron lamellar crystal structure.

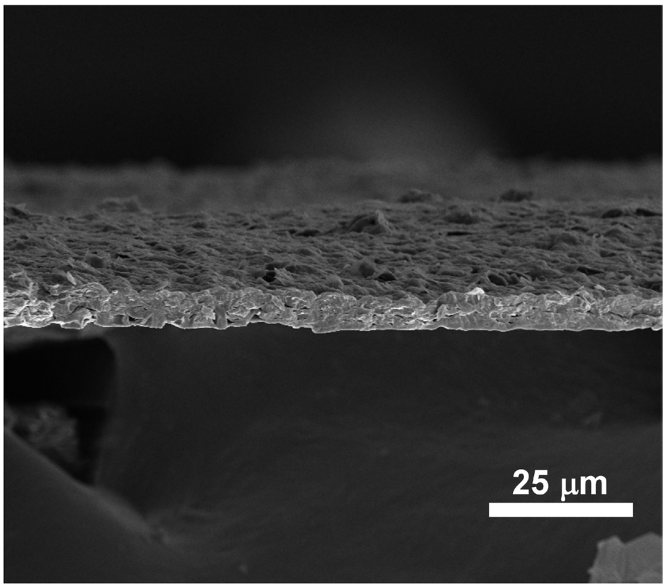

Supplementary Fig. 13 SEM image of the casted Cage-1 sensing film exhibits remarkable uniformity.

Supplementary Table 1. Proton conductivities of Cage-1 under different RH at 303 K

	**Relative humidity (RH%)**	**67**	**75**	**85**	**95**
**Cage-1**	R(Ω)	197.1	105.2	38.4	19.9
	σ (Scm^{-1})	$1.29*10^{-4}$	$2.42*10^{-4}$	$6.64*10^{-4}$	$1.28*10^{-3}$

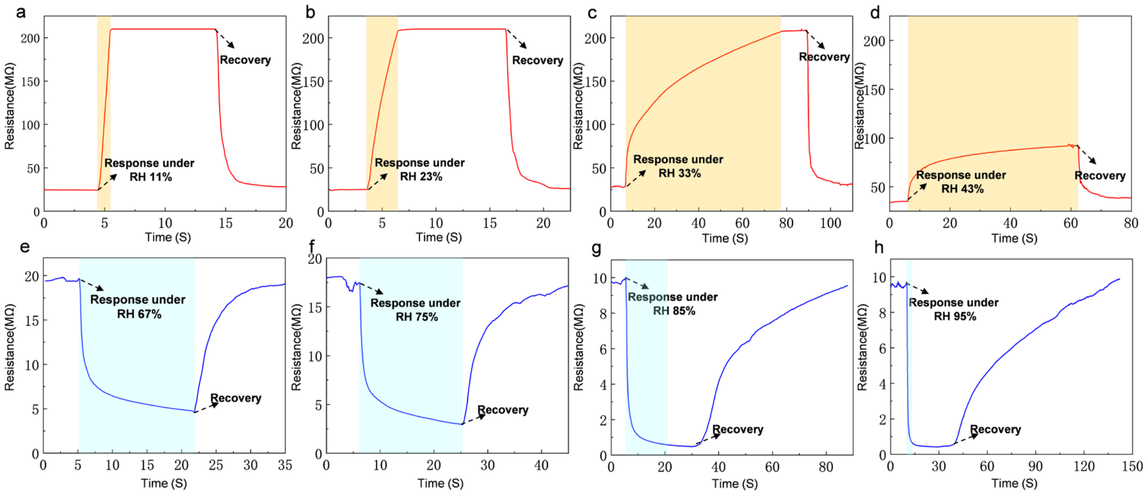

Supplementary Fig. 14 Cage-1 humidity sensing towards different saturated salt solutions conditions and recovery under indoor relative humidity.

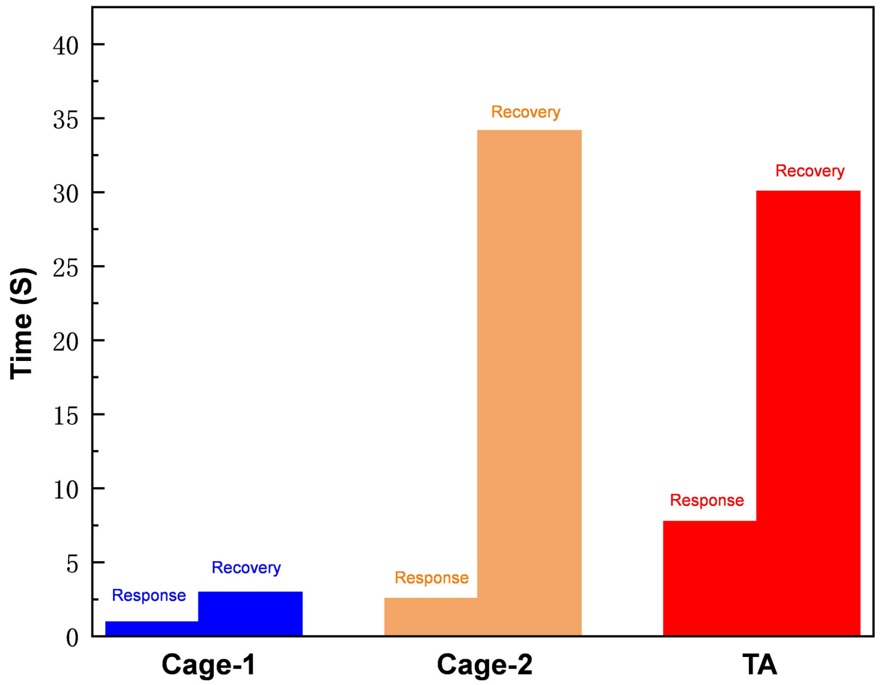

Supplementary Fig. 15 The comparison of response and recovery time of the first humidity response cycle for Cage-1, Cage-2 and TA.

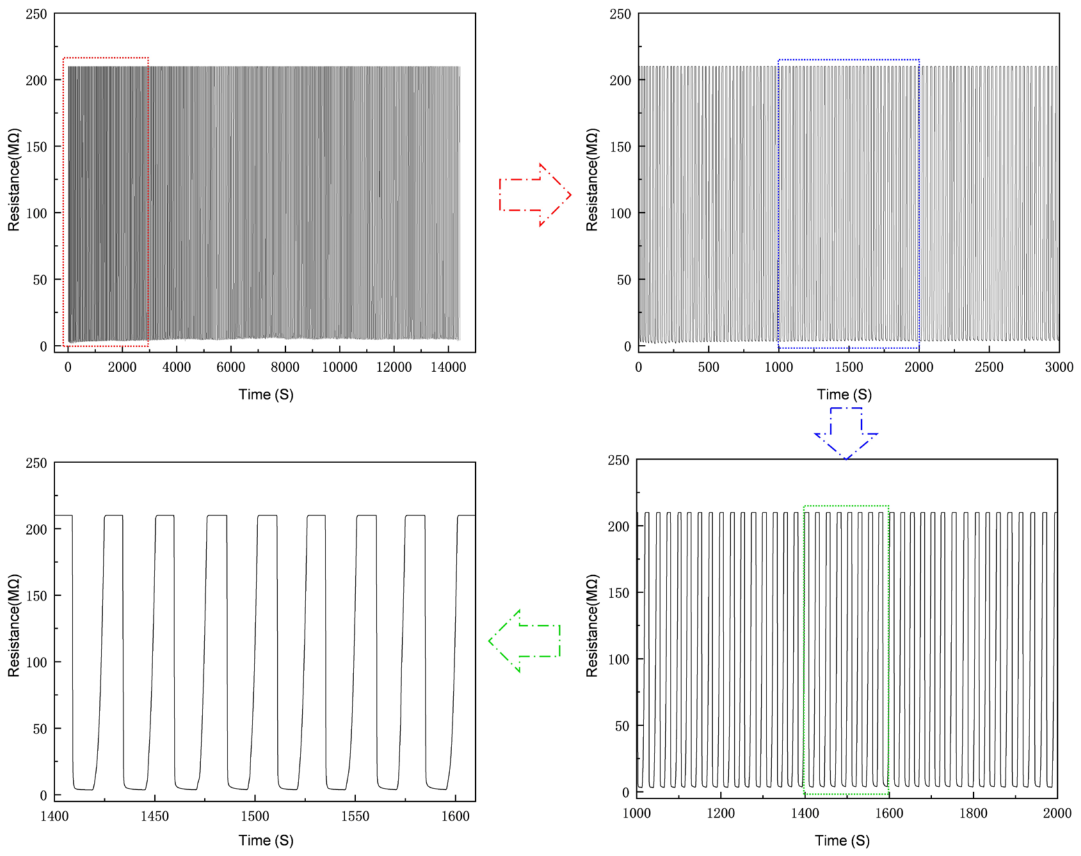

Supplementary Fig. 16 Cycle stability of the Cage-1 sensor by recording the sensor response under high humidity (95% RH) and recovery under low humidity (11% RH).

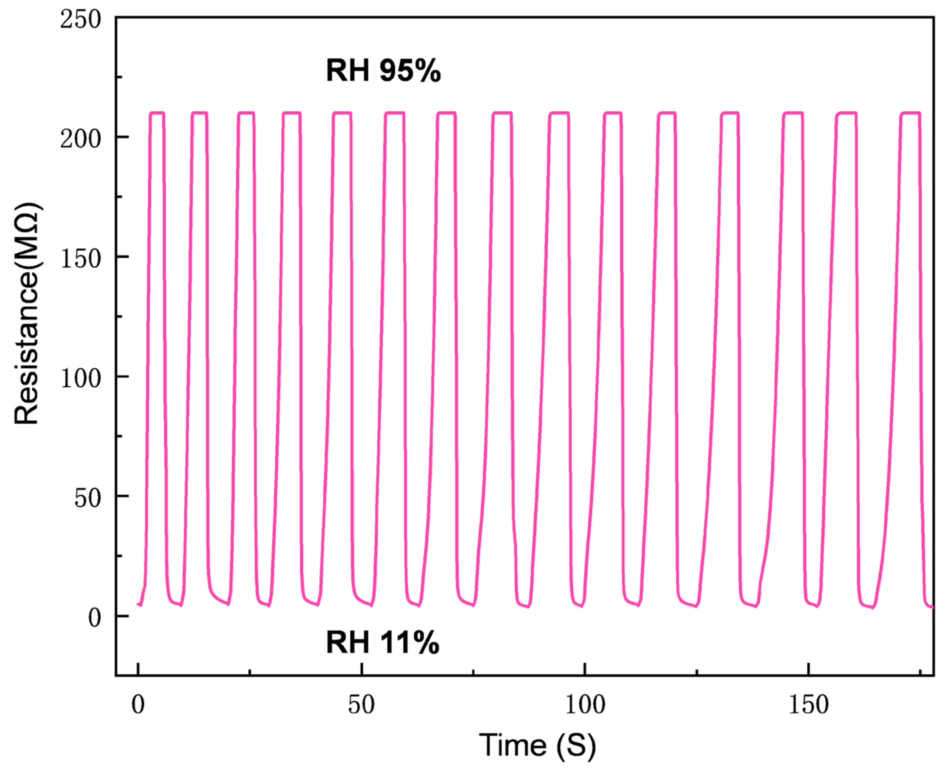

Supplementary Fig. 17 95% RH -11% RH responsive cycle stability of the Cage-1 sensor with exposure to the indoor environment with 60% RH for 3 months.

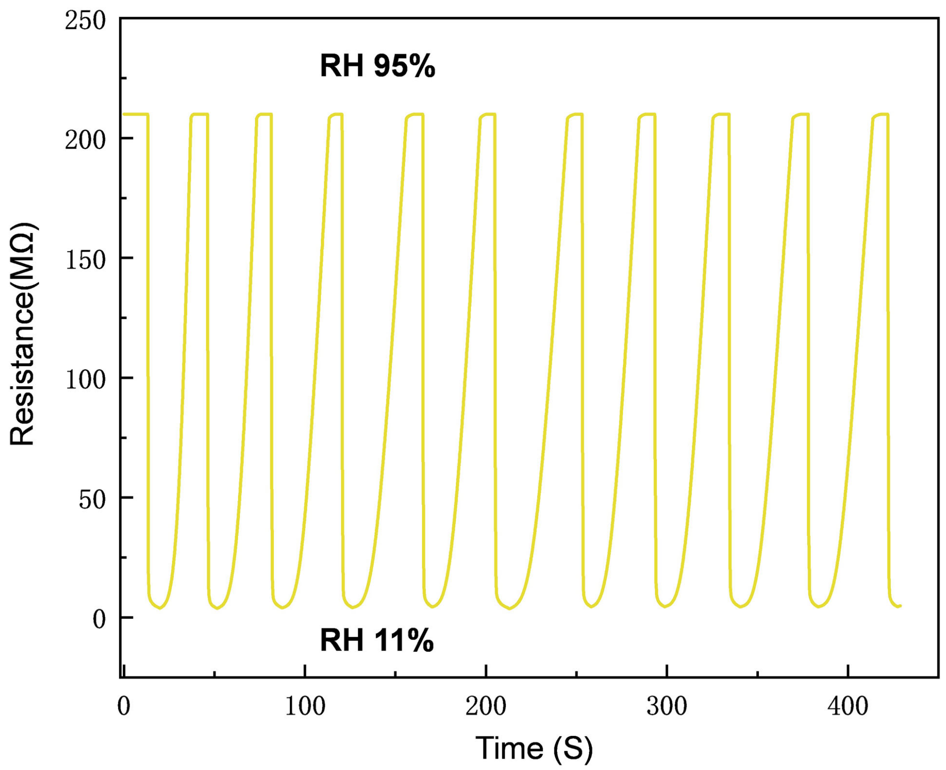

Supplementary Fig. 18 95% RH -11% RH responsive cycle stability of the Cage-1 sensor with exposure to the 95% RH for 36 hours.

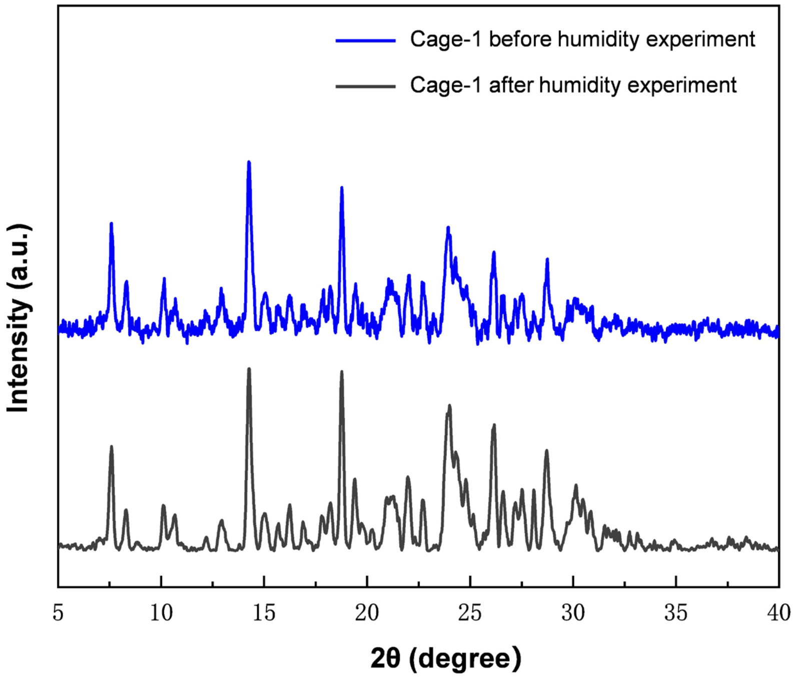

Supplementary Fig. 19 PXRD comparison of the Cage-1 before and after humidity experiments

Supplementary Table 2. Humidity sensing performances comparison of different materials.

**Material**	**Type**	**Humidity range (% RH)**	**Response time(s)**	**Recovery time(s)**	**Cyclic performance (cycles)**
Er doped ZnO nanoparticles[1]	Impedance	11-95	32	39	3
Cu doped ZnO thin film[2]	Resistive	15-95	32	47	-
KCl-doped TiO2 nanofibre[3]	impedance	11-95	3	3	-
Mg2+/Na+- doped TiO2 nanofiber[4]	impedance	11-95	2	1	10
SnO2 dodecahedral nanocrystals[5]	impedance	11-95	4	13	3
SnO2-WS2 nano-composite[6]	Resistive	11-95	-	50	5
LiCl-Pebax 2533[7]	impedance	11-95	30	80	-
SBA-15-PSS[8]	impedance	11-95	5	106	5
keratin/graphene oxide[9]	impedance	16–92	41	62	-
Graphene Oxide[10]	impedance	15–95	10.5	41	several

PEVIm-Br[11]	Resistive	11-98	6	31	4
Nanofiber of SPEEK[12]	impedance	11-98	1	25	5
PAM/cassava gum- polyol - LiBr[13]	impedance	11-98	275.6	227.0	4
PVA/CNF[14]	impedance	11-98	380	140	3
Graphene Oxide[15]	Resistive	12–97	50	421	5
PANI/NFC/PVA[16]	Capacitive	30-100	47	58	4
PEDOT: rGO-PEI/Au[17]	Resistive	11-98	20	-	<100
Graphene[18]	Resistive	1–96	0.6	0.4	3
Pyranine-rGO[19]	impedance	11-95	2	6	100
RGO-BiVO4 heterojunction[20]	impedance	11-95	3.6	18	30
Li/K-codoped 3DOM WO3[21]	impedance	11-95	15	10	5
Ag/SnO2[22]	impedance	11-95	4	6.5	2
Graphene Oxide[23]	Capacitive	10-90	15.8	-	6
[P(VDF-TrFE][24] nanocone arrays	Capacitive	50-90	3.7	3.4	3
Silk fibroin[25]	Resistive	43-95	73.1	11.3	5
MoO3[26]	Resistive	0-100	0.5	2	5
Pd/HNb3O8[27]	Resistive	30-99.9	0.2	3	50
VS2[28]	Resistive	0-100	30–40	12–50	4
WS2[29]	Resistive	11-97	12	13	4
Pt-nRGO fiber[30]	Resistive	6.1-66.4	0.064	0.508	30
TiO2 nanowire[31]	Voltage	20-90	4.5	2.8	5
MWCNTs/PAA[32]	Resistive	50-90	680	380	3
MWCNTs/ PVP[33]	Resistive	11-94	15	1.8	3
MnO2-coated CNT yarn[34]	Resistive	65-90	20	30	5
N-doped carbon spheres[35]	Impedance	9-97	19	178	3
PDDA/rGO[36]	Resistive	11-97	108–147	94–133	15
Au/GO/silica[37]	Impedance	20-90	119	125	-
15 nm Graphene Oxide[38]	Impedance	10-90	30ms	30ms	5
**Cage (This work)**	**Resistive**	**11-95**	**1**	**3**	**800**

Supplementary Table 3. Crystallographic details for Cage-2.

IDENTIFICATION CODE	Cage-2
**Empirical formula**	[C_{54} H_{85} $Cl_{5.77}$ N_8 O_6]
**Formula weight (g/mol)**	1147.02
**Temperature /K**	120.0
**Crystal system**	trigonal
**Space group**	R -3
***a*/ Å**	17.1379(10)
***b*/ Å**	17.1379(10)
***c*/ Å**	39.9405(17)
**α/°**	90
**β/°**	90
**γ/°**	120
**Volume/ $Å^3$**	10159.2(13)
**Z**	6
**ρcalcg/cm^3**	1.125
**F(000)**	3667.0
**Radiation**	MoKα (λ = 0.71073)
**reflections collected**	35282
**Independent reflections**	4610 (R_{int}= 0.0894)
**Data/restraints/para meters**	2786/127/225
**Goodness-of-fit on F2**	1.410
**Final R indexes [I>=2σ (I)]**	$R1$ = 0.1190, $wR2$ = 0.3663
**CCDC numbers**	2253396

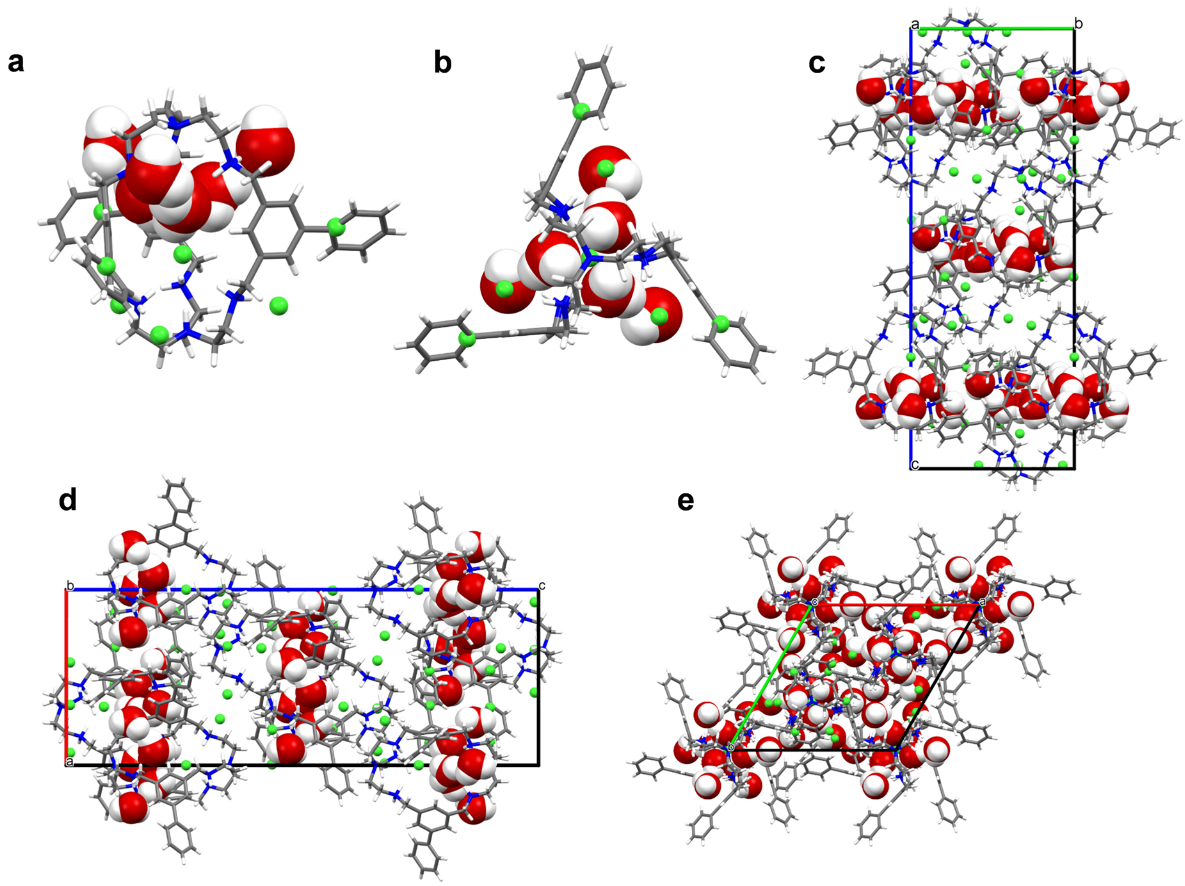

Supplementary Fig. 20 Crystal structure of Cage-2. **(a)** Side view and **(b)** top view of crystal structure of Cage-2, and its molecular packing viewed along **(c)** a, **(d)** b, and **(e)** c axis. Water molecules are displayed as a sphere, while the protonated cages are shown in stick model form and chloride anions are shown in ball stick mode. Color code: green, Cl; red, O; blue, N; white, H; grey, C.

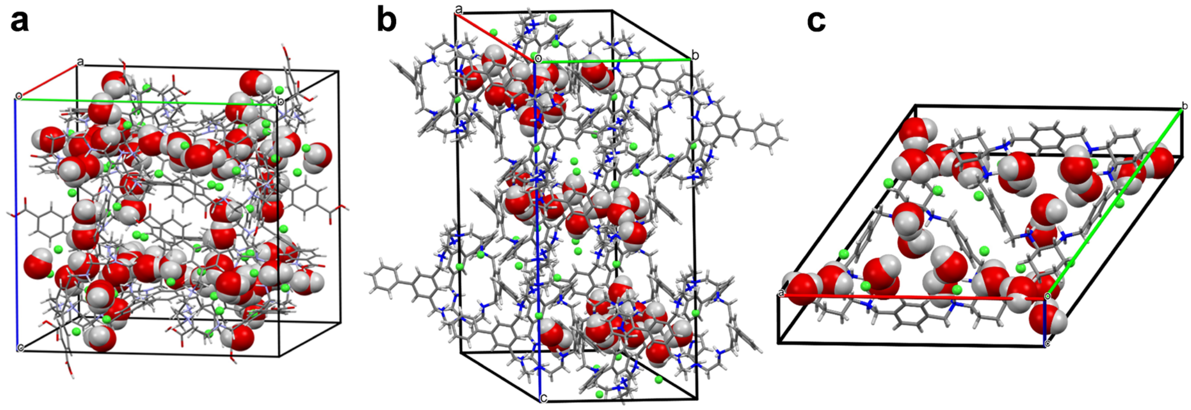

Supplementary Fig. 21 Comparison of crystal structures. Crystal cell of **(a)** Cage-1, **(b)** Cage-2 and **(c)** TA. Water molecules are displayed as a sphere, while the protonated cages are shown in stick model form and chloride anions are shown in ball stick mode. Color code: green, Cl; red, O; blue, N; white, H; grey, C.

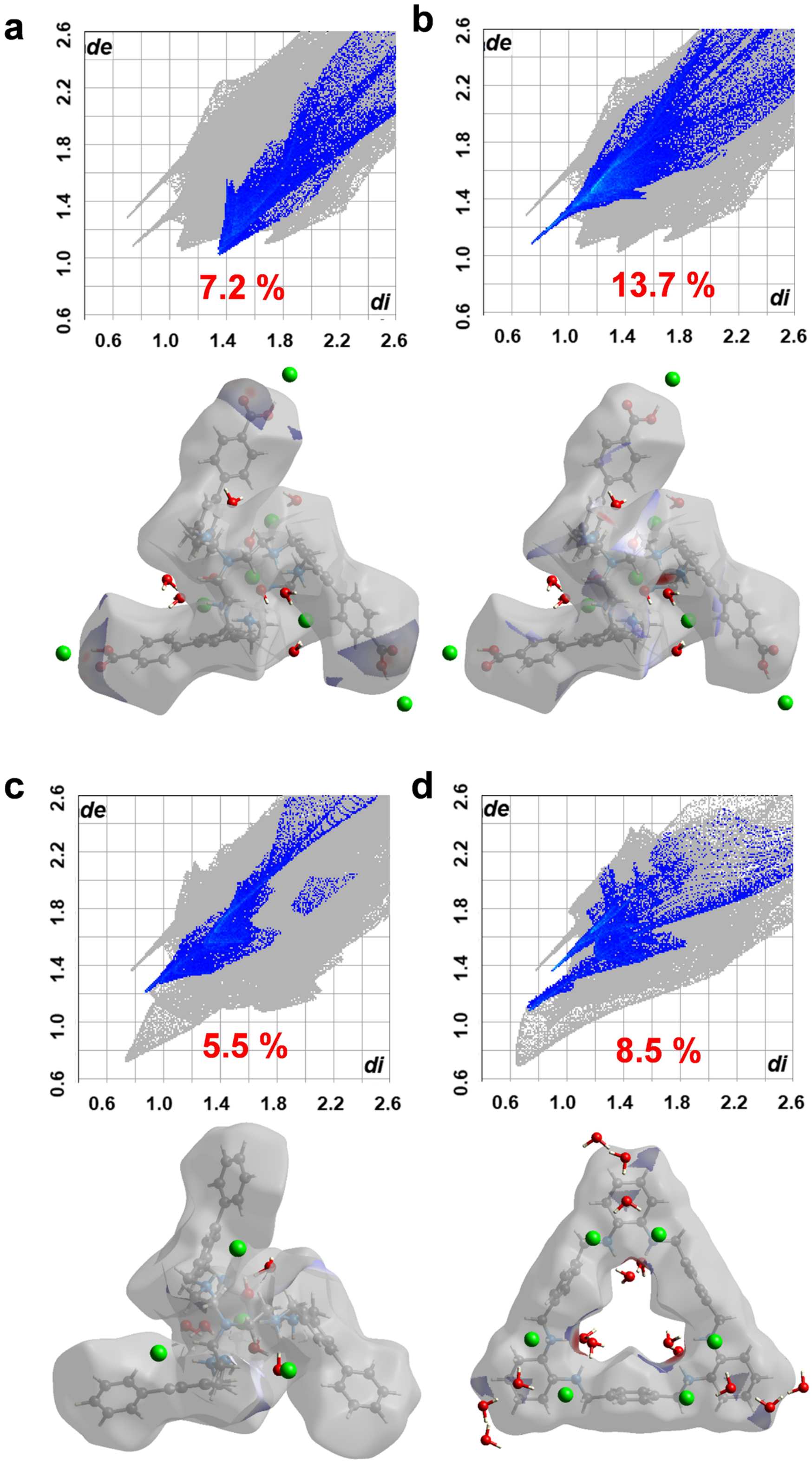

Supplementary Fig. 22 Hirshfeld surface analysis of the H···O interactions in the crystals. 2D fingerprint plots of displaying the percentage contributions of **(a)** O···H, **(b)** H···O of Cage-1, and H···O of **(c)** Cage-2 and **(d)** TA macrocycle and their Hirshfeld surface mapped with d_{norm}. X···Y: X belongs to hosts, Y belongs to the guests.

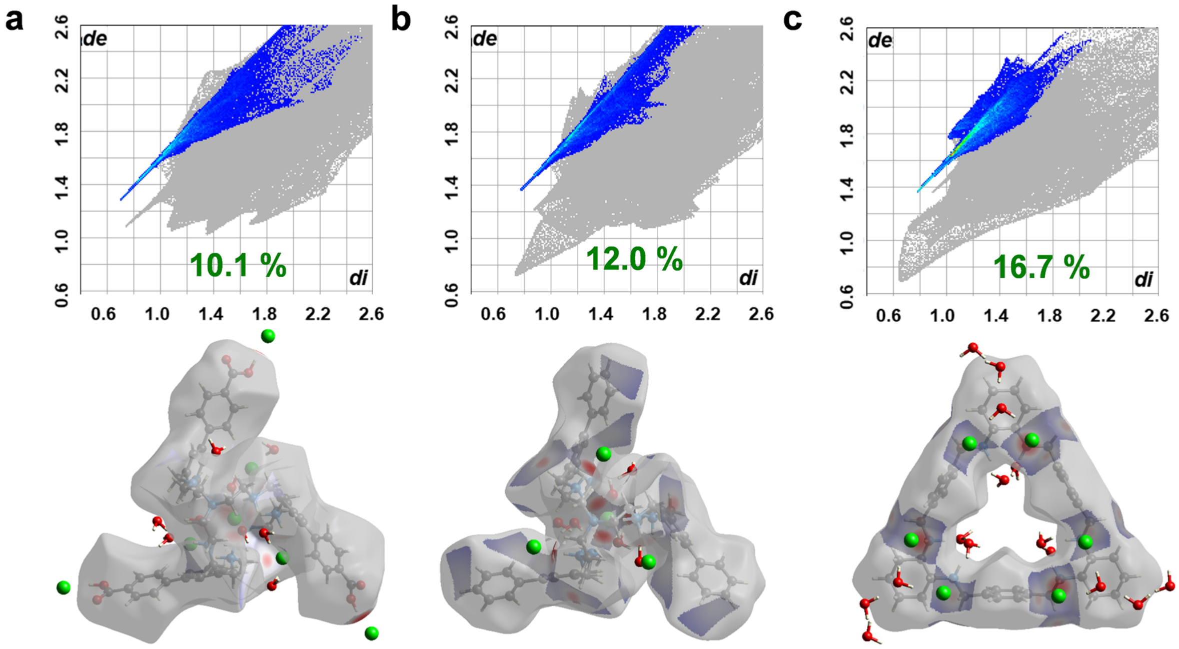

Supplementary Fig. 23 Hirshfeld surface analysis of the H···Cl interactions in the crystals. 2D fingerprint plots of displaying the percentage contributions of H···Cl of **(a)** Cage-1, **(b)** Cage-2 and **(c)** TA macrocycle and their Hirshfeld surface mapped with d_{norm}. X···Y: X belongs to host, Y belongs to the guests.

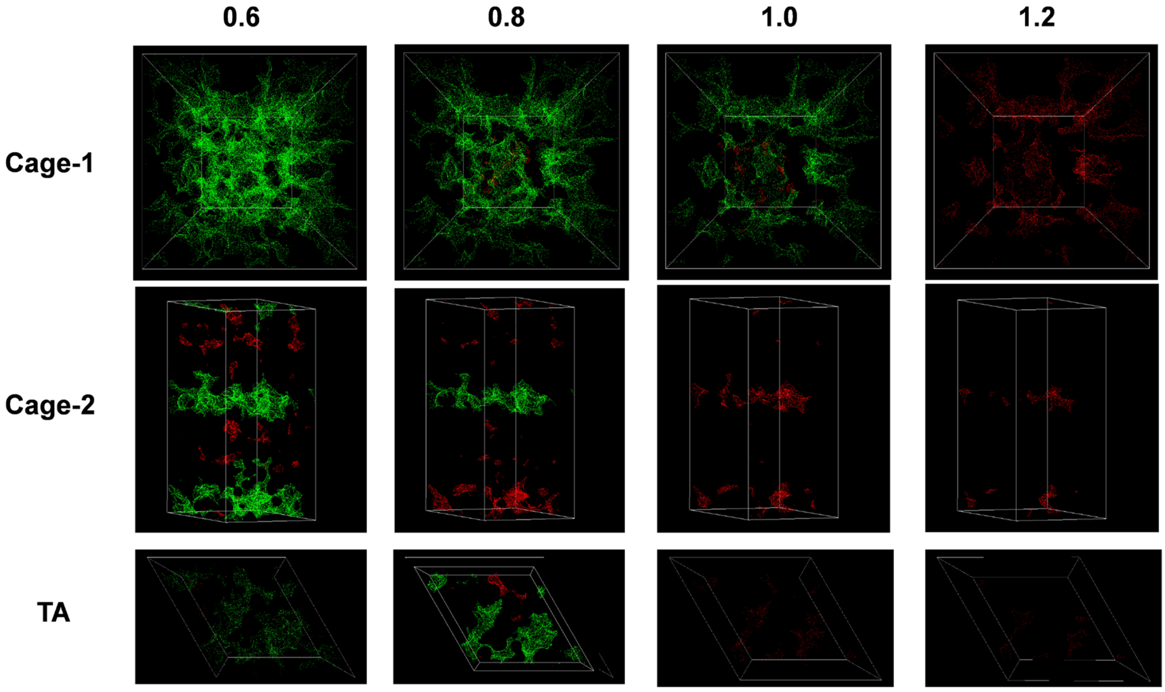

Supplementary Fig. 24 Structural analysis of the crystals. Interconnected (green) and isolated (red) voids space of the cage Cage-1, Cage-2 and macrocycle TA considering relative probes with radius as shown (Å).

Supplementary Table 4. Proton conductivities at different temperatures under 85% RH

	Temperature (K)	303	313	323	333	343
**Cage-1**	R(Ω)	38.4	37.4	36.5	34.9	33.2
	σ (Scm^{-1})	6.64*10^{-4}	6.81*10^{-4}	6.98*10^{-4}	7.3*10^{-4}	7.67*10^{-4}
**Cage-2**	R(Ω)	972.9	547.7	298.1	204.6	95.2
	σ (Scm^{-1})	2.62*10^{-5}	4.65*10^{-5}	8.55*10^{-5}	1.25*10^{-4}	2.68*10^{-4}
**TA**	R(Ω)	35960	18049	4783	1443	552
	σ (Scm^{-1})	7.08*10^{-7}	1.41*10^{-6}	5.33*10^{-6}	1.77*10^{-5}	4.61*10^{-5}

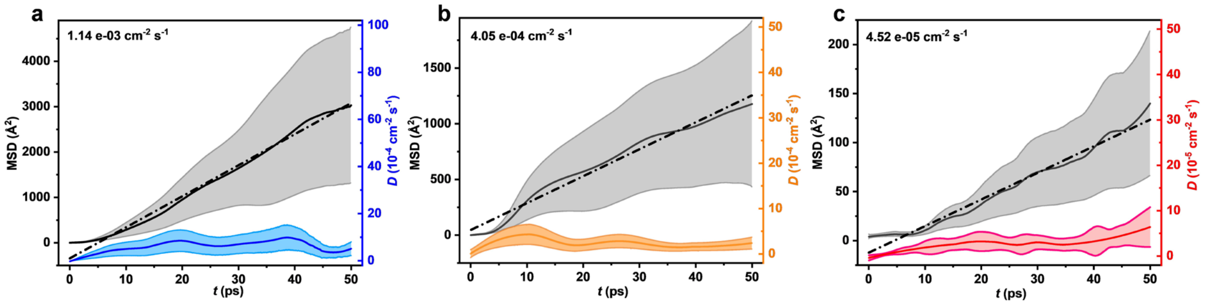

Supplementary Fig. 25 Comparison of average MSDs and diffusion coefficients (*D*$_{water}$). Average MSDs and *D*$_{water}$ of water molecules for **(a)** Cage-1, **(b)** Cage-2, and **(c)** TA, the upper and lower bounds of MSDs and *D*$_{water}$ denoting their standard errors.

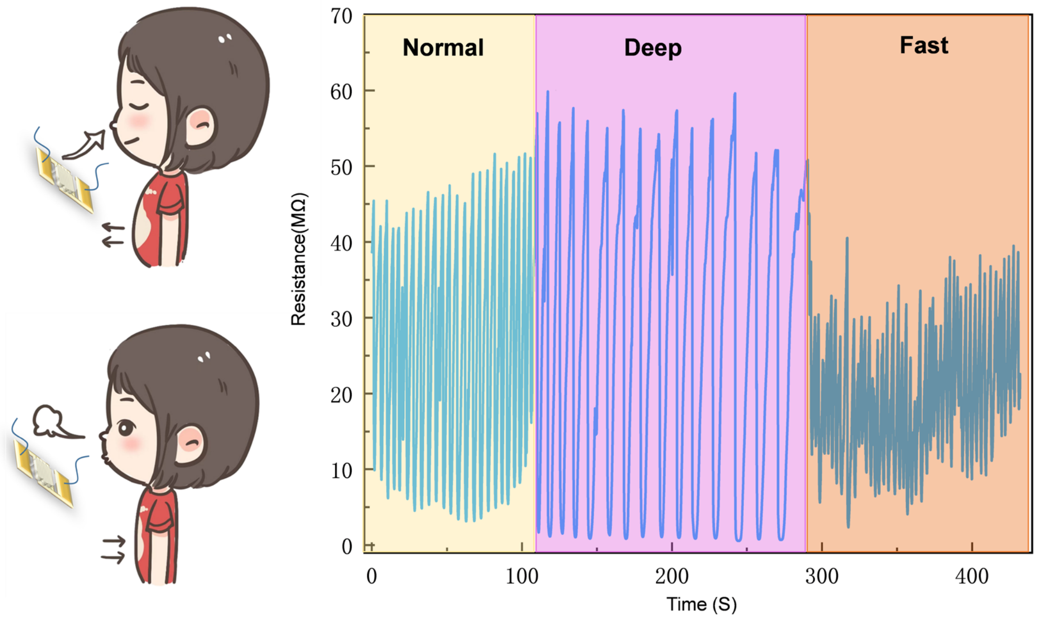

Supplementary Fig. 26 Touchless human respiration monitor based on the cage humidity sensor.

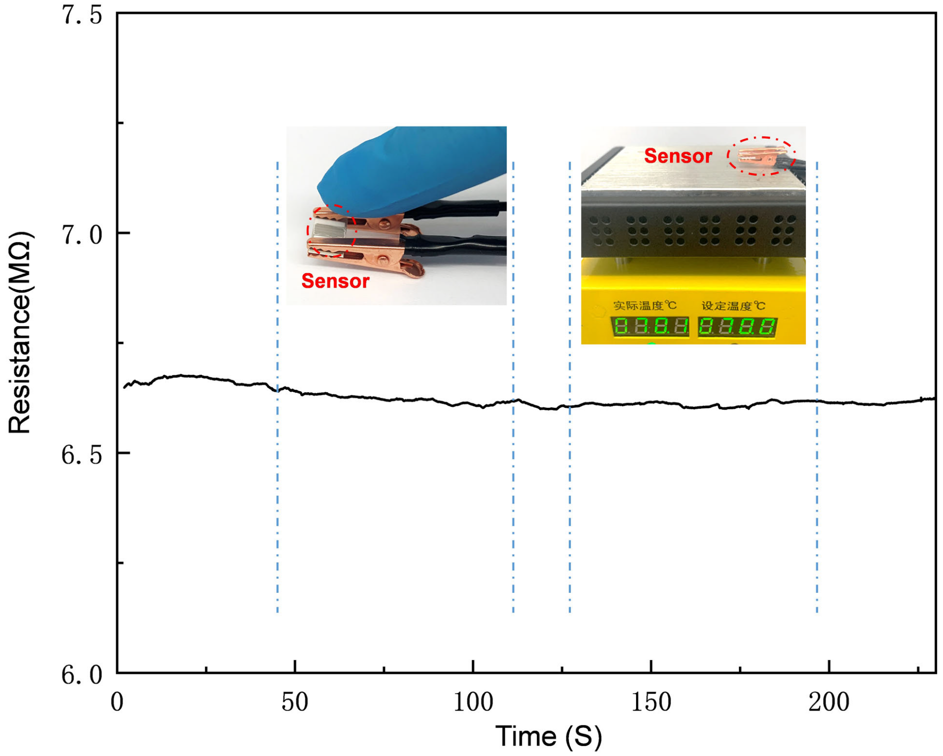

Supplementary Fig. 27 The resistance change of the cage humidity sensor under the gloved finger or a hot metal surface.

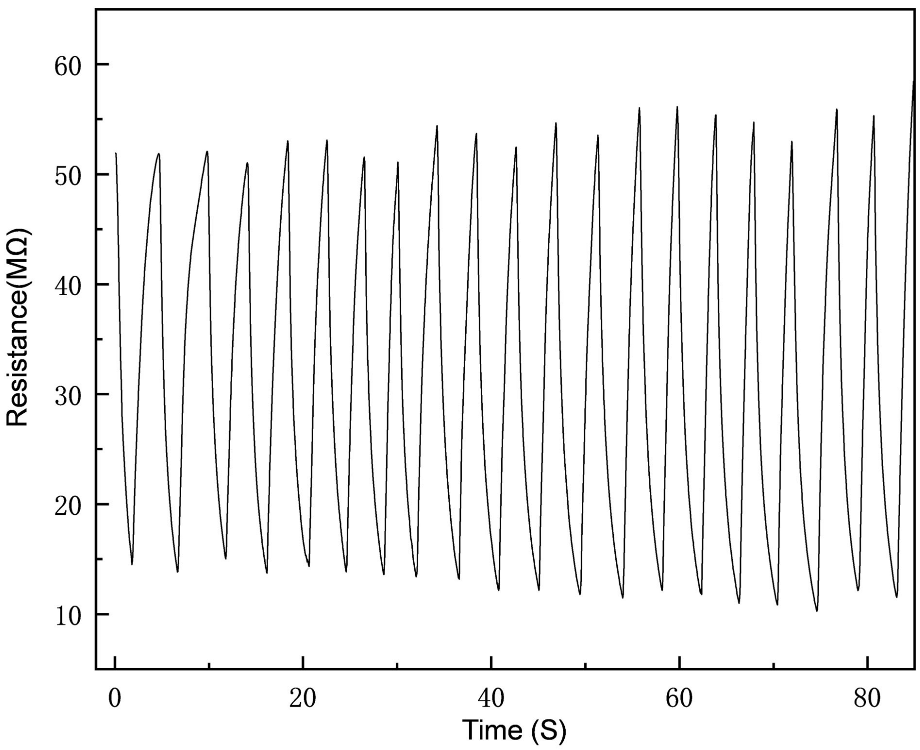

Supplementary Fig. 28 R–t curves of the cage fingertip humidity sensor after abrasion test.

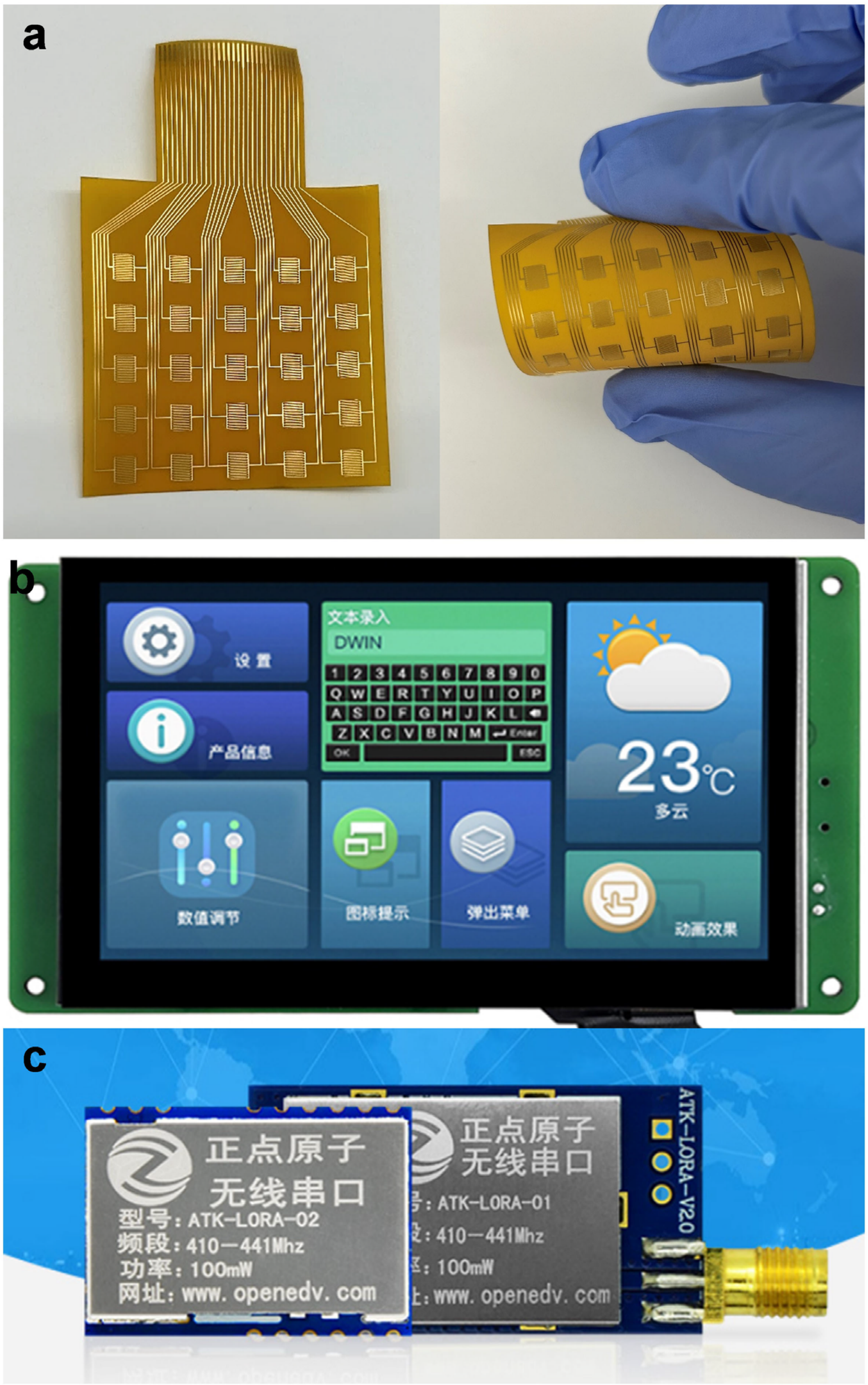

Supplementary Fig. 29 The main modules of the contactless device. (a) 100um Flexible Interdigitated Electrode Array: 1. Substrate material: Pl, thickness of 0.25mm; 2. Dimensions: 50mm*74mm; 3. Number of units: 5*5; 4. Unit structure: Finger width: 100um; spacing: 100um; finger length: 3mm; number of finger pairs: 11 pairs; 5. Metal layer structure: Cu/Au, with thicknesses of 12 um and 25nm respectively. **(b)** Display Screen: 5-inch capacitive touch intelligent serial screen with IPS wide-angle screen and resolution of 800*480. **(c)** Wireless module: 433MHz wireless serial transmission and reception RF module.

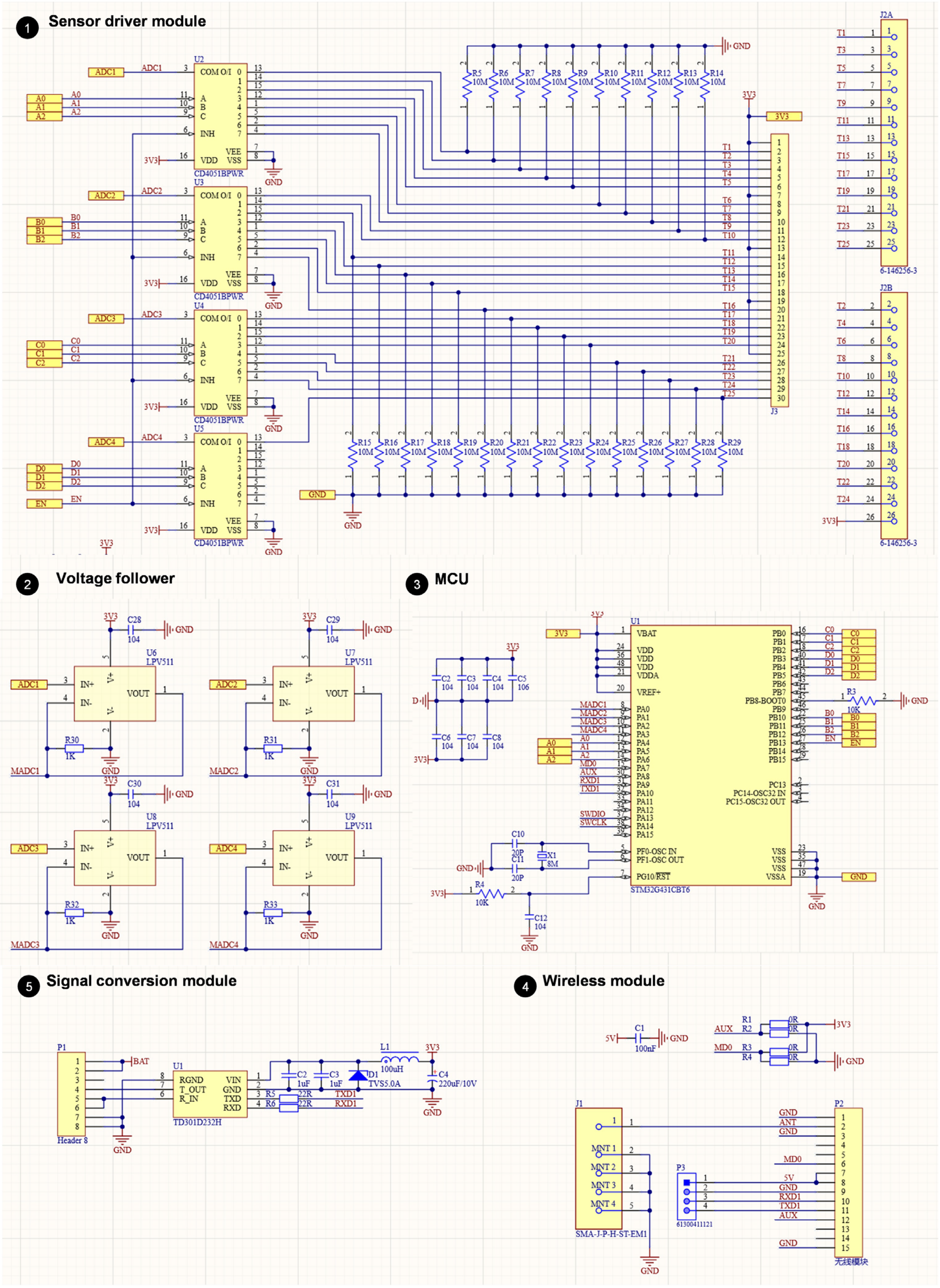

Supplementary Fig. 30 Circuit diagram for main modules of the touchless control screen.

Supplementary references

1. Zhang, M., Zhang, H., Li, L., Tuokedaerhan, K. & Jia, Z. Er-enhanced humidity sensing performance in black ZnO-based sensor. *Journal of Alloys and Compounds* **744**, 364–369 (2018).
2. Misra, S. K. & Pandey, N. K. Study of activation energy and humidity sensing application of nanostructured Cu-doped ZnO thin films. *J. Mater. Res.* **31**, 3214–3222 (2016).
3. Qi, Q., Feng, Y., Zhang, T., Zheng, X. & Lu, G. Influence of crystallographic structure on the humidity sensing properties of KCl-doped TiO2 nanofibers. *Sensors and Actuators B: Chemical* **139**, 611–617 (2009).
4. Zhang, H. *et al.* Mg2+/Na+-doped rutile TiO2 nanofiber mats for high-speed and anti-fogged humidity sensors. *Talanta* **79**, 953–958 (2009).
5. Feng, H. *et al.* Three-dimensional hierarchical SnO2 dodecahedral nanocrystals with enhanced humidity sensing properties. *Sensors and Actuators B: Chemical* **243**, 704–714 (2017).
6. Chen, Y. *et al.* Humidity sensing properties of the hydrothermally synthesized WS2-modified SnO2 hybrid nanocomposite. *Applied Surface Science* **447**, 325–330 (2018).
7. Liang, S. *et al.* Highly sensitive humidity sensors based on LiCl–Pebax 2533 composite nanofibers via electrospinning. *Sensors and Actuators B: Chemical* **208**, 363–368 (2015).
8. Zhao, H. *et al.* Organic-inorganic hybrid materials based on mesoporous silica derivatives for humidity sensing. *Sensors and Actuators B: Chemical* **248**, 803–811 (2017).
9. Hammouche, H., Achour, H., Makhlouf, S., Chaouchi, A. & Laghrouche, M. A comparative study of capacitive humidity sensor based on keratin film, keratin/graphene

oxide, and keratin/carbon fibers. *Sensors and Actuators A: Physical* **329**, 112805 (2021).

10. Bi, H. *et al.* Ultrahigh humidity sensitivity of graphene oxide. *Sci Rep* **3**, 2714 (2013).

11. Wang, L., Duan, X., Xie, W., Li, Q. & Wang, T. Highly chemoresistive humidity sensing using poly(ionic liquid)s. *Chem. Commun.* **52**, 8417–8419 (2016).

12. Li, X., Zhuang, Z., Qi, D. & Zhao, C. High sensitive and fast response humidity sensor based on polymer composite nanofibers for breath monitoring and non-contact sensing. *Sensors and Actuators B: Chemical* **330**, 129239 (2021).

13. Liang, Y. *et al.* Humidity Sensing of Stretchable and Transparent Hydrogel Films for Wireless Respiration Monitoring. *Nano-Micro Lett.* **14**, 183 (2022).

14. Ding, Q. *et al.* Stretchable, self-healable, and breathable biomimetic iontronics with superior humidity-sensing performance for wireless respiration monitoring. *SmartMat* **4**, e1147 (2023).

15. Pang, Y. *et al.* Wearable humidity sensor based on porous graphene network for respiration monitoring. *Biosensors and Bioelectronics* **116**, 123–129 (2018).

16. Anju, V. P., Jithesh, P. R. & Narayanankutty, S. K. A novel humidity and ammonia sensor based on nanofibers/polyaniline/polyvinyl alcohol. *Sensors and Actuators A: Physical* **285**, 35–44 (2019).

17. Zhang, R., Peng, B. & Yuan, Y. Flexible printed humidity sensor based on poly(3,4-ethylenedioxythiophene)/reduced graphene oxide/Au nanoparticles with high performance. *Composites Science and Technology* **168**, 118–125 (2018).

18. Smith, A. D. *et al.* Resistive graphene humidity sensors with rapid and direct electrical readout. *Nanoscale* **7**, 19099–19109 (2015).

19. Chen, Z. *et al.* One-Step Fabrication of Pyranine Modified- Reduced Graphene Oxide with Ultrafast and Ultrahigh Humidity Response. *Sci Rep* **7**, 2713 (2017).

20. Wu, Z. *et al.* Development of a rGO-BiVO $_4$ Heterojunction Humidity Sensor with Boosted Performance. *ACS Appl. Mater. Interfaces* **13**, 27188–27199 (2021).

21. Wang, Z. *et al.* Humidity-Sensing Performance of 3DOM WO $_3$ with Controllable Structural Modification. *ACS Appl. Mater. Interfaces* **10**, 3776–3783 (2018).

22. Tomer, V. K. & Duhan, S. A facile nanocasting synthesis of mesoporous Ag-doped SnO 2 nanostructures with enhanced humidity sensing performance. *Sensors and Actuators B: Chemical* **223**, 750–760 (2016).

23. Lan, L. *et al.* One-step and large-scale fabrication of flexible and wearable humidity sensor based on laser-induced graphene for real-time tracking of plant transpiration at bio-interface. *Biosensors and Bioelectronics* **165**, 112360 (2020).

24. Niu, H. *et al.* Ultrafast-response/recovery capacitive humidity sensor based on arc-shaped hollow structure with nanocone arrays for human physiological signals monitoring. *Sensors and Actuators B: Chemical* **334**, 129637 (2021).

25. Zheng, Y. *et al.* A Flexible Humidity Sensor Based on Natural Biocompatible Silk Fibroin Films. *Adv Materials Technologies* **6**, 2001053 (2021).

26. Yang, J. *et al.* Flexible Smart Noncontact Control Systems with Ultrasensitive Humidity Sensors. *Small* **15**, 1902801 (2019).

27. Lu, Y. *et al.* Highly stable Pd/HNb $_3$ O $_8$ -based flexible humidity sensor for perdurable wireless wearable applications. *Nanoscale Horiz.* **6**, 260–270 (2021).

28. Feng, J. *et al.* Giant Moisture Responsiveness of VS $_2$ Ultrathin Nanosheets for Novel

Touchless Positioning Interface. *Advanced Materials* **24**, 1969–1974 (2012).

29. Pawbake, A. S., Waykar, R. G., Late, D. J. & Jadkar, S. R. Highly Transparent Wafer-Scale Synthesis of Crystalline WS $_2$ Nanoparticle Thin Film for Photodetector and Humidity-Sensing Applications. *ACS Appl. Mater. Interfaces* **8**, 3359–3365 (2016).
30. Choi, S. *et al.* Nitrogen-Doped Single Graphene Fiber with Platinum Water Dissociation Catalyst for Wearable Humidity Sensor. *Small* **14**, 1703934 (2018).
31. Shen, D. *et al.* Self-Powered, Rapid-Response, and Highly Flexible Humidity Sensors Based on Moisture-Dependent Voltage Generation. *ACS Appl. Mater. Interfaces* **11**, 14249–14255 (2019).
32. Lee, J., Cho, D. & Jeong, Y. A resistive-type sensor based on flexible multi-walled carbon nanotubes and polyacrylic acid composite films. *Solid-State Electronics* **87**, 80–84 (2013).
33. Pan, X. *et al.* Effective Enhancement of Humidity Sensing Characteristics of Novel Thermally Treated MWCNTs/Polyvinylpyrrolidone Film Caused by Interfacial Effect. *Adv Materials Inter* **3**, 1600153 (2016).
34. Jung, D., Kim, J. & Lee, G. S. Enhanced humidity-sensing response of metal oxide coated carbon nanotube. *Sensors and Actuators A: Physical* **223**, 11–17 (2015).
35. Cunha, B. B., Greenshields, M. W. C. C., Mamo, M. A., Coville, N. J. & Hümmelgen, I. A. A surfactant dispersed N-doped carbon sphere-poly(vinyl alcohol) composite as relative humidity sensor. *J Mater Sci: Mater Electron* **26**, 4198–4201 (2015).
36. Zhang, D., Tong, J. & Xia, B. Humidity-sensing properties of chemically reduced graphene oxide/polymer nanocomposite film sensor based on layer-by-layer nano self-assembly. *Sensors and Actuators B: Chemical* **197**, 66–72 (2014).

37. Su, P.-G., Shiu, W.-L. & Tsai, M.-S. Flexible humidity sensor based on Au nanoparticles/graphene oxide/thiolated silica sol–gel film. *Sensors and Actuators B: Chemical* **216**, 467–475 (2015).

38. Borini, S. *et al.* Ultrafast Graphene Oxide Humidity Sensors. *ACS Nano* **7**, 11166–11173 (2013).
