# Supplementary material for: Smart touchless human–machine interaction based on crystalline porous cages
Source: Nat Commun. 2024 Feb 21;15:1575. doi: 10.1038/s41467-024-46071-8 (PMC10881501; doi:10.1038/s41467-024-46071-8)
Supplement: Supplementary file 3 — Description of additional supplementary files [file 41467_2024_46071_MOESM3_ESM.pdf]

Description of Additional Supplementary Files

Supplementary Movie 1 : A movie showing that the resistance of the Cage-1 humidity sensor reduced when the finger approached to the sensor surface, and increased rapidly when the finger moved away.

Supplementary Movie 2 : A movie showing that the robust durability of the Cage-1 sensor through an abrasion test, subjecting the sensing film to vigorous rubbing by a finger dozens of times.

Supplementary Movie 3 : A movie showing that the continuous glide gesture monitoring of the touchless control screen.

Supplementary Movie 4 : A movie showing that the intermittent tap monitoring of the touchless control screen.

Supplementary Movie 5 : A movie showing that the line click monitoring of the touchless control screen.

Supplementary Movie 6 : A movie showing that the application of the touchless electronic screen to write the number "1".

Supplementary Movie 7 : A movie showing that the application of the touchless electronic screen to write the number "2".

Supplementary Movie 8 : A movie showing that the application of the touchless electronic screen to write "王".

Supplementary Movie 9 : A movie showing that the screen displaying a happy face when the password matches.

Supplementary Movie 10 : A movie showing that the screen displaying a sad face when the password is incorrect.
